# Genome Sequences of *Pseudomonas oryzihabitans* Phage POR1 and *Pseudomonas aeruginosa* Phage PAE1

**DOI:** 10.1128/genomeA.01515-15

**Published:** 2016-06-16

**Authors:** Zoe A. Dyson, Robert J. Seviour, Joseph Tucci, Steve Petrovski

**Affiliations:** a La Trobe Institute of Molecular Sciences, Bendigo, Victoria, Australia; b Department of Physiology, Anatomy and Microbiology, La Trobe University, Bundoora, Victoria, Australia

## Abstract

We report the genome sequences of two double-stranded DNA siphoviruses, POR1 infective for *Pseudomonas oryzihabitans* and PAE1 infective for *Pseudomonas aeruginosa*. The phage POR1 genome showed no nucleotide sequence homology to any other DNA phage sequence in the GenBank database, while phage PAE1 displayed synteny to *P. aeruginosa* phages M6, MP1412, and YuA.

## GENOME ANNOUNCEMENT

*Pseudomonas aeruginosa* and *Pseudomonas oryzihabitans* are opportunistic pathogens that have developed resistance to a wide range of antimicrobials (1–5). Here we describe two phages, PAE1 and POR1 infective for *P. aeruginosa* and *P. oryzihabitans*, respectively.

Phage PAE1 was isolated from a wastewater treatment plant in Bendigo (Victoria, Australia), and formed plaques on *P. aeruginosa* strain PAO9505 lawn cultures (6). It is a member of the family *Siphoviridae* with a B2 capsid morphotype. Its genomic DNA was prepared and sequenced as described previously (7). A minimum sequence coverage of 50 times was obtained. Genomes were assembled using gsAssembler (v2.6) (Roche Applied Science, Indianapolis, IN, USA), and open reading frames (ORFs) were predicted with Glimmer (v3.02) and manual annotations (8). Homology and conserved domain searches were performed using BLASTp (http://blast.ncbi.nlm.nih.gov/Blast.cgi), and the presence of tRNA sought with the tRNAscan-SE program (9).

Genomic analysis revealed that the 62,818-bp genome of the *Siphovirus* PAE1 displays between 93% and 97% nucleotide sequence identity to that of phages MP1412 (61,167 bp; accession no. JX131330), M6 (59,446 bp; accession no. DQ163916), and YuA (58,663 bp; accession no. AM749441) over a coverage of 92%, 93%, and 89%, respectively (10–12). DNA sequence comparisons reveal recombination events have most probably occurred leading to DNA deletions and/or insertions, and generating sequence variations seen between PAE1 phage and its closest relatives. A total of 88 open reading frames (ORFs) were detected, and no tRNAs seen. The PAE1 genome has a modular architecture, with regions associated with DNA packaging (*orf1-2*), virion morphogenesis/host lysis (*orf7-orf31*), and DNA replication/maintenance identified (*orf37-orf79*). The presence of a phage integrase (*orf63*) suggests PAE1 might be capable of a lysogenic existence (10, 13). The integrase gene appears highly conserved in PAE1 and its relative phages MP1412, M6, and YuA and together with the high level of sequence similarity to *Pseudomonas aeruginosa* genomes, suggests these phages too are probably temperate.

Phage POR1 was isolated from a sample obtained from the Nambour (QLD, Australia) wastewater treatment plant, and formed plaques on lawn plates of *P. oryzihabitans* strain J81P. Phage POR1 is also a member of the *Siphovirus* family*,* possessing a B1 capsid morphotype. The POR1 genome was sequenced as described above obtaining a 107 times coverage and revealing the genome size of 55,349 bp, sharing no nucleotide sequence homology with any sequence in GenBank database. Given that many of the predicted gene products shared similarity to those encoded by their bacterial hosts, it is predicted that POR1 is a prophage. Sequencing and restriction endonuclease profiling revealed that both PAE1 and POR1 had circularly permuted genome, but whether terminal repeats or headful packaging is used is unknown.

### Nucleotide sequence accession numbers.

Genome sequences of phages PAE1 and POR1 have been deposited in GenBank under accession numbers KT734862 and KT716399, respectively.
